# Short-term prediction of threatening and violent behaviour in an Acute Psychiatric Intensive Care Unit based on patient and environment characteristics

**DOI:** 10.1186/1471-244X-11-44

**Published:** 2011-03-18

**Authors:** Arne E Vaaler, Valentina C Iversen, Gunnar Morken, John C Fløvig, Tom Palmstierna, Olav M Linaker

**Affiliations:** 1 Department of Neuroscience, Faculty of Medicine, Norwegian, University of Science and Technology, Trondheim, Norway; 2 Division of Psychiatry, Department of Research and Development, St Olavs University Hospital, Trondheim, Norway; 3 Division of Psychiatry, Department Østmarka, St Olavs University Hospital, Trondheim, Norway; 4 Social and Forensic Psychiatry Program, Stockholm Centre for Psychiatric Research and Education, Karolinska Institutet/Stockholm County Council Health Care Provision, Stockholm, Sweden; 5 St. Olav's University Hospital, Forensic Dept. and Research Centre Brøset, Trondheim, Norway

## Abstract

**Background:**

The aims of the present study were to investigate clinically relevant patient and environment-related predictive factors for threats and violent incidents the first three days in a PICU population based on evaluations done at admittance.

**Methods:**

In 2000 and 2001 all 118 consecutive patients were assessed at admittance to a Psychiatric Intensive Care Unit (PICU). Patient-related conditions as actuarial data from present admission, global clinical evaluations by physician at admittance and clinical nurses first day, a single rating with an observer rated scale scoring behaviours that predict short-term violence in psychiatric inpatients (The Brøset Violence Checklist (BVC)) at admittance, and environment-related conditions as use of segregation or not were related to the outcome measure Staff Observation Aggression Scale-Revised (SOAS-R). A multiple logistic regression analysis with SOAS-R as outcome variable was performed.

**Results:**

The global clinical evaluations and the BVC were effective and more suitable than actuarial data in predicting short-term aggression. The use of segregation reduced the number of SOAS-R incidents.

**Conclusions:**

In a naturalistic group of patients in a PICU segregation of patients lowers the number of aggressive and threatening incidents. Prediction should be based on clinical global judgment, and instruments designed to predict short-term aggression in psychiatric inpatients.

**Trial registrations:**

NCT00184119/NCT00184132

## Background

Threatening and violent behaviour by psychiatric inpatients are major concerns in psychiatric practice [1,2]. Aggression has negative consequences for patients and staff. Some studies indicate an increasing frequency [3,4]. Reduction of severity and incidence of threatening and violent incidents are important in order to improve quality of care in psychiatric facilities. Prediction of violence is consequently important in order to initiate preventive measures. Risk factors and accuracy of predictions in different psychiatric settings have been extensively described [5].

In Psychiatric Intensive Care Units (PICUs) and emergency services violent incidents are frequent and short-term predictions of violence important [6]. Patients in these settings present in severe crisis often complicated by behavioural dyscontrol, substance use, and multiple axis 1 diagnoses [7]. Prediction and prevention of aggression in emergency services patients could thus potentially be based on numerous data drawn from individual medical and social history, treatment conditions, behaviours and psychopathology. A history of violence in medical records, and a diagnosis of schizophrenia or substance abuse are known predictors for violence [5,8]. However, in the emergency setting with acute admittances of unknown patients these factors are often unknown and thereby clinically irrelevant. These are indications that such predictors have more limited value in emergency clinical settings compared to community or forensic settings [5,8-10]. In acute settings short-term prediction may be more accurate based on global clinical judgment [9-11], or rating scales specifically developed to assess short-term prediction of aggressive incidents [12]. In a retrospective study from a PICU population Bjørkdahl et al demonstrated that nurses can predict violence to a high degree for the next 24 hours with three times daily assessments using the Brøset Violence Checklist (BVC) [12].

Differences in methods, designs, outcome measures and settings make it difficult to compare studies of short-term prediction of violence. The weaknesses in most designs are underreporting of incidents, lack of correction for therapeutic interventions, and a disregard of the influence from the treatment environment [5,9]. Environment-related conditions like extent of segregation of behaviourally disturbed patients are believed to influence rate of aggressive incidents [13].

Rating scales specifically developed to measure frequency and severity of violent or threatening incidents, a prospective research design, and control of environment-related conditions and treatment factors are believed to minimize the problems in research methods.

The frequency of threatening and violent incidents differs with the stages of psychiatric disorders [14]. The highest frequencies of such incidents are reported in acute stages of in-patient hospital stay which indicate the first few days in emergency settings.

The aims of the present prospective study were to investigate clinically relevant patient and environment-related predictive factors for threatening and violent incidents the first three days in a psychiatric acute emergency population based on evaluations at admittance. These patient-related conditions are actuarial data (age, gender, admission status and diagnosis), assessments of symptoms, function and behaviour, therapeutic interventions the first day, global clinical impression assessed by physician on duty, global clinical impression assessed by treating nurses, and predictive properties from a single BVC rating at admittance; while the environment-related condition is the use of the PICU as a segregation area or not.

## Methods

### Population

The psychiatric department at St. Olavs University Hospital, Trondheim, Norway has a catchment area of 140.000 inhabitants. About 700 patients above 18 years with acute psychiatric conditions are admitted each year. Norwegian acute psychiatric emergency services are publicly funded and available to everyone. All the patients in the catchment area are admitted to this department. Acute admissions to other psychiatric hospitals occur only if inhabitants temporarily reside outside the catchment area when the need for acute admittance arises.

### Setting

The acute department consists of two ordinary closed emergency wards each with a PICU area with 4 beds. The patients were admitted to the acute ward with most free capacity. One ward was used for the study, and the patients excluded from the study were admitted to the other ward.

The physician on duty evaluated all the patients acutely admitted to the ward. Patients assessed to be in need of PICU were admitted to the PICU area and included in the study except those with dementia, mental retardation or autism to a severe degree, and patients not speaking Norwegian or English. They were excluded at evaluation before entering the PICU area and admitted to the other ward.

The study ward consists of an ordinary closed acute ward area (310 m^2^) and a PICU area (190 m^2^). The main entrance leads to the ordinary area of the ward. In the end of the corridor a locked door separates the PICU area from the ordinary area. The PICU area consists of two wings with two single patient rooms in each. The patients stay mostly in the wings together with nurses, and contact with other patients is limited. The PICU area thus provides segregation from other persons. A sketch of the ward is published previously [15].

Data from the present study stems from two different inclusion periods in the PICU with background data published previously [15,16]. The two samples were comparable for all measurements at inclusion [15]. In the first inclusion period the entrance door to the PICU area was permanently locked and the doors inside the PICU leading to the wings were kept permanently closed (inclusion1). In the second period the entrance door to the PICU area was removed and the doors inside the PICU were permanently open (inclusion 2). These two conditions made it possible to compare two different methods of organizing a PICU [15]. Inclusion 1 was a completely segregated PICU-condition, while inclusion 2 was a condition giving the patients opportunity to choose between a certain level of isolation and calmness by staying in the patient room/wing of the PICU or move to the main part of the ward ensuing exposure to other patients, more staff-members and increased amounts of sensory and emotional stimuli. The clinical staffs were similar in these inclusion periods. There was no significant difference in occupation of PICU-beds between inclusion 1 and 2.

The environment-related condition in the inclusions thus was the use of the PICU as a segregation area or not [15]. The difference between the inclusions as a possible predictive factor for SOAS-R incidents is the parameter "Segregation".

### Instruments

Symptoms, general psychopathology, function and behaviour were assessed with a single rating on the first day in the PICU with the Positive and Negative Syndrome Scale (PANSS) for schizophrenia [17] with time criterion the last 24 hours, the Global Assessment Scale Split version (GAF-S), and the Brøset Violence Checklist (BVC) [18]. GAF-S is based on DSM-4's GAF [19] and is a two-item scale measuring global symptoms (GAF-S-Symptoms) and functioning (GAF-S-Function) separately. BVC is a six-item observer-rated scale scoring behaviours that predicts imminent violence in psychiatric inpatients [20,21]. BVC predictions are traditionally performed three times daily [20,21] as opposed to the present study evaluating predictive properties for the next three days from a single assessment at admittance. Since psychometric properties of The PANSS used in an emergency setting with time criterion last 24 hours is not previously tested, two trained ward nurses evaluated this in a separate pre-study. Through scorings of 3 video-taped patient interviews [22] and assessments of 12 consecutively admitted acute emergency patients, the ward nurses demonstrated excellent inter-rater reliability for total PANSS sum, sums of positive (Pearson's r = 0.96), negative (r = 0.84) and general subscales (r = 0.87), as well as the 30 single items.

Violent or threatening incidents were recorded with the Staff Observation Scale-Revised (SOAS-R) [23,24]. The SOAS-R severity score ranges from 0-22 points with higher scores indicating greater severity [25]. A SOAS-R score ≥ 9 indicates a serious incident [12,24].

Therapeutic- and control steps taken and nurses' observations were coded on a 23-item checklist. These therapeutic steps and observations included for instance "frequency of testing out and pushing limits", "intensity of testing out and pushing limits", "need to set limits", all prescribed medication, side effects, formal restrictions (restrictions regarding visits and telephone), staff contact time, use of newspapers, and visits from relatives. Depending on the type of item each were scored on scales 0-4 (0 = not present, 4 = very much) or 0-1 (0 = not used, 1 = used). Specially trained unit nurses did all the ratings. The first rating with the items "frequency of testing out and pushing limits", "intensity of testing out and pushing limits", and "need to set limits" was used as a possible predictor after an initial, short observation of the patient's behaviour at admittance and right after entrance to the PICU in order to evaluate whether the experienced staff's assessments of the patients' immediate behaviour could predict SOAS-R incidents for the rest of the study period.

At admittance the physician on duty evaluated the patients' need for PICU on a scale with scorings 1-4 (1 representing no need to 4 representing absolute need). Four categories of reasons for admittance to PICU were noted (1: Patient's own wish, 2: Need of close observation from diagnostic or medical reasons, 3: Reduction of stimuli, or 4: Control of behaviour). If more than one reason was present, the physician indicated the dominating category. "Physician's prediction" is an index defined by giving the patients with category 4 reason for admittance the scorings on "patients' need" of PICU, and the rest of the patients value 0. "Physicians prediction" therefore has scorings 0-4 with increasing value indicating increasing assumed probability for violent or threatening incidents.

The patients were systematically examined for substance use at admittance, at evaluation with ward psychiatrist the first weekday after admittance, and at discharge from PICU. In the first period (November 13 2000 to March 25 2001) (n = 56), urine samples were analysed on clinical suspicion of substance use. In the second period (October 1 2001 to March 21 2002) (n = 62), all admitted patients had urine- and blood samples taken within a few hours after admission.

Diagnoses according to ICD-10 Diagnostic criteria for research [26] were set by consensus in the department's staff, including at least three specialists in psychiatry of whom at least two personally had examined the patient.

### Statistical Analyses

All data were analysed using the Statistical Package for the Social Sciences (version 11.0). Demographic and clinical variables were described using means and frequencies. Independent t-tests were used to compare differences between groups. Multiple logistic regression forced-entries were performed to examine the extent of the predictor variables' associations with SOAS-R incidents. The variable SOAS-R was categorised as incidents and non-incidents. Relative risk (RR) was calculated with Fischer's exact test and a generalized mixed model with Poisson distribution of SOAS-R incidents. The missing values were replaced with the mean score for each item. Statistical significance was defined as a two-tailed p < 0.05.

### Ethics

The study was approved by "The Regional Medical Research Ethics Committee, Central Norway."

## Results

### Characteristics of the sample

In the two inclusion periods 56 and 62 patients were included. Mean length of stay in the PICU was 5.6 days (SD 0.6). One patient was excluded due to senile dementia. The number of males/females was 66/52 with mean age 36.3 years (SD 14.7). Fifty-seven (48.3%) were involuntarily admitted. The number of patients in each category of reasons for admittance to PICU were: Patient's own wish 3 (2.5%); Need of close observation from diagnostic or medical reasons 51 (43.2%); Reduction of stimuli 23 (19.5%); and Control of behaviour 40 (33.9%). The patients admitted to PICU due to "control of behaviour" had a mean need for stay = 3 (3; indicating probable need for segregation and 4; absolute need for segregation). The first three days a total of 3 (inclusion 1) (the PICU-condition with complete segregation) and 19 (inclusion 2) (the PICU-condition without complete segregation) (RR 5.72, p < 0.01(Poisson distribution), 95% CI: 1.69-19.33) violent or threatening incidents were recorded among 3 (inclusion 1) (complete segregation) and 10 (inclusion 2) patients (11%) (RR 3.01, ns (p = 0.08) (Fischer's exact test), 95% CI: 0.81-20.10). The effect for segregation indicates that patients who were not segregated were 3.0 times more likely to engage in a violent incident than those who were segregated.

The mean SOAS-R severity score for these incidents was 10.1 (SD 14.7). The distribution of scores of SOAS-R -incidents and no SOAS-R-incidents, were found to have a mean of 0.11 (SD = .031). The number of serious incidents defined as SOAS-R severity score ≥ 9 was 16. The incidents with severity scores <9 were all verbal threats directed towards staff. Mechanical restraints (belts) were used twice in each inclusion period.

The main diagnoses were F 00-09 (organic mental disorders) 6, F 10-19 (substance abuse) 24, F 20-29 (schizophrenia) 45, F 30-39 (mood disorders) 22 and F 40+ (other mental disorders) 21. Nine patients (coded F 20-29) fulfilled criteria for both schizophrenia and substance abuse diagnoses. The prevalence of different substances used by the patients in the present department is published previously [27].

### Selection of variables for analyses

Due to the limitations in the number of variables in the multiple regression analysis, the individual variables of possible interest were analysed for possible contribution to SOAS-R incidents with chi square tests for categorical and Student's T-tests for continuous data. PANSS total, PANSS subscales, GAF-S, medication, side effects, "frequency of testing out and pushing limits", "intensity of testing out and pushing limits", "need to set limits", gender and age did not contribute significantly. The items BVC, "Segregation" and "Physicians prediction" items were selected for subsequent multiple logistic regression forced-entry analyses (Table 1). A further analysis was carried out to examine the items that predicted SOAS-R incidents. Diagnoses of schizophrenia and substance abuse were used as independent variables since earlier studies have shown that aggressiveness has been related to schizophrenia and substance abuse [28-30]. The proportions of patients with these diagnoses were 36.4% and 38.1% respectively.

**Table 1:** Comparison of patients with SOAS incidents (n = 13) and without SOAS Incidents (n = 105).

	SOAS incidents Mean/SD	Non-SOAS incidents Mean/SD	Statistical significance
Total PANSS^α^	85.5(25.3)	72.4(21.4)	NS

GAF-S^β^	24.5(12.6)	32.6(12.9)	NS

GAF-F^β^	23.4(9.3)	33.8(12.4)	NS

BVC^γ^	2.54(1.9)	.60(.87)	P = .002

Side effects			NS
Extrapyramidal	.00 (.00)	.02(.19)	
Acathisia	.00(.00)	.05(.29)	

Frequency of testing out and pushing limits	1.46(1.12)	.50(.92)	NS

Need to set limits	1.54(1.05)	.53(1.97)	NS

Intensity of testing out and pushing limits	1.92((1.55)	.50(.98)	NS

Segregation	1.77(.43)	1.50(.50)	P = .041

Diagnosis of schizophrenia	.46(.51)	.37(.48)	NS

Diagnosis of substance abuse	.07(.27)	.21(.41)	NS

Physician's prediction	3.85(.55)	2.70(.29)	P = .039

Medication			NS
Hypnotics and sedatives	.31(.48)	.37(.48)	
Anti-depressive	.08(.27)	.18(.38)	
Anti-epileptic	.00(.00)	.16(.37)	
Neuroleptic	.38(.50)	.31(.46)	

SD = standard deviation. NS = not significant. Significance level p ≤ 0.05.

^α ^The Positive and Negative Syndrome Scale for schizophrenia. Scoring range 30-210.

^β ^The Global Assessment of Functioning Scale - Split version. Scoring range 1-100.

^γ ^The Brøset Violence Checklist. Scoring range 0-6.

A multiple logistic regression forced-entry analysis was performed on SOAS-R as outcome variable and the five predictors: BVC, "Physicians prediction", "Segregation", and diagnosis of schizophrenia or substance abuse. The results are given in Table 2. The size of R^2 ^(58%) indicates that the model contributes powerfully to the prediction of the SOAS-R incidents or non-incidents. The variables BVC, "Physicians prediction", and "Segregation" contribute significantly to predict SOAS-R incidents. The equation did not find statistically significant associations between SOAS-R (incidents or non-incidents) and a diagnosis of schizophrenia or substance abuse. The Hosmer-Lemeshow test was non-significant indicating that the fit of the model was good (p = 0.55).

**Table 2:** Multiple logistic regression predicting SOAS-R incidents.

Predictors	95% CI for exp b			
		
	B (SE)	Lower	Exp b	Upper
BVC	1.07**	1.42	2.91	5.91
"Segregation"	2.23*	1.32	9.37	66.31
Physicians prediction	1.66*	1.20	5.28	23.16
Diagnosis of substance abuse	-1.59	0.033	0.03	1.26
Diagnosis of schizophrenia	0.26	0.24	1.29	6.51

Note: R^2 ^= .57 (Hosmer&Lemeshow), .29 (Cox & Snell R.), .58 (Negelkerke R.) Model X^2 ^(5) = 40.10 *p < 0.05, **p < 0.01

## Discussion

The present study gives evidence for a positive effect of segregation quantitatively determined in relation to other variables. We are not aware of the presence of such evidence so far. This finding is highly relevant for both clinical practice and the design of psychiatric wards. Effects of ward space and architecture are sparsely studied. Palmstierna et al found that patients with schizophrenia were more likely to be aggressive in a crowded ward [31]. In a second study the same authors did not find a decline in the frequency of aggression in spite of a reduction of the number of beds by 50% [32]. Nijman et al were unable to document a decline in aggressive incidents after extending space in a ward [33]. In the present study the number of beds and the space were identical in the two inclusions [15]. This finding indicates that an important factor in reducing aggressive incidents is the separation of single patients or patient groups in the ward, not the physical space in terms of square meters per patient.

Our data predicting short-term violent and aggressive incidents in a PICU are in accordance with previous studies from acute wards. Generally the predictive values from actuarial data are limited. The global clinical evaluation "Physicians prediction" from physician on duty, and the observer-rated scale scoring behaviours predicting imminent violence in psychiatric inpatients (BVC), were more suitable for predicting short-term violent and threatening incidents in the PICU setting. Since BVC is based on observer rated scorings of behaviour, the present study demonstrates that experienced staff members in acute settings are able to globally predict short-term violence in their patient populations.

We found no association between SOAS-R ratings and psychopathology measured by PANSS total, PANSS subscales, and GAF-S. This finding is similar to Swett et al's [34]. Steinert et al. in contrast found that scorings on the seven-item PANSS-positive scale correlated significantly with the number of threatening or aggressive incidents in a sample of acutely admitted in-patients [35]. Findings from studies using the Brief Psychiatric Rating Scale (BPRS) [36] or PANSS are contradictory. Using the full scale PANSS is time consuming, but this systematic questioning discloses important aspects of symptoms and makes the staff able to take these into account in therapy. This may lower the number of violent or threatening incidents, and make conclusions from different studies difficult [16].

The observer rated instrument BVC is based on reports of the most frequent behaviours observed prior to violent incidents, and it assesses the presence or absence of the six behavioural states confusion, irritability, boisterous behaviour, verbal threatening, physical threatening, and attacking objects [20]. It has demonstrated satisfactory properties in forensic and acute settings [21,37], and now in a PICU setting. The instrument is short, practical and easy to administer in routine care. Systematic uses of standardised instruments like BVC give staff opportunities to focus on preventive measures towards limited numbers of high-risk patients.

Involuntary admission status did not predict SOAS-R incidents in the present study. This finding is contrary to Nijman et al.'s who found a history of involuntary admission to be a predictor of aggressive behaviour [10]. This may partly be due to different criteria for involuntary admissions. Some countries (e.g. Dutch law [10]) allow forced hospitalisation only when a patient's behaviour constitutes a direct and clear danger to the patient or others. Norwegian law extends this concept to allow involuntary admissions in other cases of severe mental illness based on the need of treatment.

Diagnoses of substance use or schizophrenia are reported to be predictive factors for aggressive incidents [5,28-30]. This was not supported by the present data. Assessment of substance use is difficult and under-reporting is a problem. In the present study substance abuse was extensively assessed. Our study indicates that presence of substance use diagnoses do not facilitate threatening and violent behaviour among patients in a PICU setting [38].

Several studies with different interventions have been conducted to assess the effects of preventive measures on aggressive incidents [39]. Conclusions are difficult to draw due to shortcomings in the research designs like lack of control conditions, possible under-reporting of aggressive incidents and staffs' awareness of their wards being objects of research. There are also indications that systematic monitoring of aggressive incidents with for instance SOAS-R, increases the staffs' awareness of risk factors eventually leading to a decrease in numbers of incidents. Nijman et al [39] compared the effects of several possible aggressive incidents reducing interventions in a closed psychiatric admission ward with two similar control wards. The main results were a significant reduction of aggressive incidents in all the three wards. The reduction in the intervention ward and control wards were 62% and 43%, a difference that turned out to be non-significant. The present study indicates that global experience in staff and structured instruments may help identify patients where preventive measures should be considered. These measures should include physical separation of these high-risk patients from the others.

This study has weaknesses. "Physicians prediction" is an index composed of the physician on duty's global impression of the patients need and reason for admittance to PICU. This is not a validated instrument, but reflects the main outcome of what goes on in the mind of the experienced clinician. The nurse-rated 23-item checklist "Therapeutic- and control steps taken and nurses' observations" has similar shortcomings. The SOAS-R incidents are few, but comparable to other studies. The mean severity score of the incidents is moderate.

The study sample is a consecutive, highly-selected sample of acutely admitted patients assessed in a PICU. The methods and facilities used for emergency psychiatry differ between countries [40]. This special PICU have similarities to what Bowers names "open area seclusion" [40]. Different selections in different facilities may give other results. However, the principles of stimulus reduction and segregation from other patients are similar to other segregation settings. This may indicate that our study generally illustrates the effects seen also in traditional seclusion settings.

Segregation of patients raises ethical and legal questions. During the last years there have emerged new legislations, recommendations, court cases and professional guidelines to control the use of coercive measures in psychiatry. The recurring message in all of these guidelines is the need to practice caution when applying segregation in the form of seclusion [41]. The present segregation setting represents an effective alternative to seclusion with the patients staying mostly in the wings together with nurses [15].

The strengths of the study are numerous. First of all this is a prospective design in a naturalistic patient population from a defined catchment area. We have used robust validated instruments assessing symptoms, general psychopathology, function, behaviours and end-point measure. The routine screening for substance abuse was comprehensive. Therapeutic and control steps taken the first day were assessed. The degree of potential under-reporting of aggressive or threatening incidents is limited due to the prospective design and daily prompting for registrations. Finally the influence of the physical environment is incorporated in evaluation.

## Conclusions

In the psychiatric emergency setting the procedure of segregation of single patients predicts lowered numbers of short-term aggressive incidents. Predictions at admittance should further be based on a global clinical evaluation from the physician at admittance, or rating with instruments designed to predict short-term aggression in psychiatric in-patients like the Brøset Violence Checklist.

## Competing interests

The authors declare that they have no competing interests.

## Authors' contributions

AEV, VCI, GM, JCF and OML conceived and designed the study. VCI and AEV coordinated the study including the inclusion of patients. TP analyzed and interpreted the data. All authors helped to draft the manuscript, and all authors read and approved the final version.

## Pre-publication history

The pre-publication history for this paper can be accessed here:

http://www.biomedcentral.com/1471-244X/11/44/prepub
